# Imatinib Resistance in Chronic Myeloid Leukemia Is Associated with Distinct Metabolic, Oxidative, and Autophagic Adaptations in K562-Derived Cell Sublines

**DOI:** 10.3390/biomedicines14102198

**Published:** 2026-09-28

**Authors:** Ezzahra Rachid, Meriem Lahmouad, Rawane Bellemrrabet, Youssef Aboussaleh, Loubna Amahdar, Oualid Abboussi

**Affiliations:** 1 Science & Engineering of Biomedicals, Biophysics and Health Laboratory, Higher Institute of Health Sciences, Hassan First University of Settat, Settat 26002, Morocco; r.bellemrrabet.doc@uhp.ac.ma (R.B.); loubna.amahdar@uhp.ac.ma (L.A.); 2 Physiology and Physiopathology Team, Faculty of Sciences, Mohammed V University in Rabat, Rabat 10000, Morocco; o.abboussi@um5r.ac.ma; 3 Biology and Health Laboratory, Department of Life Sciences, Faculty of Sciences, University Ibn Tofail, B.P. 133, Kenitra 14000, Morocco; meriemlahmouad4@gmail.com (M.L.); youssef.aboussaleh@uit.ac.ma (Y.A.)

**Keywords:** chronic myeloid leukemia, imatinib resistance, metabolic reprogramming, oxidative phosphorylation, glycolysis, oxidative stress, reactive oxygen species, autophagy

## Abstract

**Background/Objectives:** Chronic myeloid leukemia (CML) is a hematopoietic stem cell malignancy initiated by the BCR::ABL1 oncoprotein. Although tyrosine kinase inhibitors (TKIs) have revolutionized CML therapy, resistance remains a major clinical challenge. Emerging evidence suggests that metabolic reprogramming contributes to TKI resistance, yet its relationship with oxidative stress and autophagy remains poorly characterized. **Methods**: Metabolic profiles, redox-related parameters, and autophagy markers were assessed in parental K562, imatinib-sensitive K562-S, and imatinib-resistant K562-R cells. Metabolic activity was first assessed via the MTT assay to determine the half-maximal inhibitory concentration (IC_50_) for each line, which was subsequently used as the treatment dose. Glycolytic and mitochondrial functions were profiled via Seahorse extracellular flux analysis under basal conditions. Oxidative stress was assessed via reactive oxygen species (ROS) and nitric oxide (NO) levels, and the levels of autophagy markers (p62 and LC3-I/II) were measured via Western blotting. **Results**: Imatinib reduced metabolic activity in K562 and K562-S cells (IC_50_ = 0.445 and 0.137 µM, respectively), whereas K562-R cells retained high metabolic activity across the tested range (0.1–1 µM), which was consistent with a resistant phenotype. Compared with parental K562 cells, both K562-S and K562-R cells presented reduced glycolytic activity. K562-S cells displayed greater basal respiration than K562-R cells, which presented reduced basal respiration and proton leakage but preserved spare respiratory capacity, indicating a distinct mitochondrial respiratory phenotype. Imatinib increased ROS in K562 and K562-S cells but not in K562-R cells, whereas NO increased in all three lines. Autophagy-related alterations occurred in K562 and K562-S cells, while K562-R cells presented minimal changes. **Conclusions**: Imatinib resistance involves distinct metabolic and autophagic adaptations across K562-derived cell lines. These pathways may represent potential therapeutic targets warranting further investigation to overcome TKI resistance in CML.

## 1. Introduction

Chronic myeloid leukemia (CML) is a clonal myeloproliferative neoplasm characterized by increased and unregulated growth of predominantly myeloid cells in the bone marrow and their accumulation in the blood [1,2,3,4]. The defining feature of CML is the Philadelphia chromosome, resulting from a reciprocal translocation between chromosomes 9 and 22 (t(9;22) (q34; q11)). This translocation results in the formation of a chimeric gene, BCR::ABL1, which encodes a constitutively active tyrosine kinase [5,6].

CML accounts for approximately 15–20% of all leukemia cases in adults, with an annual incidence of approximately 1–2 cases per 100,000 individuals [7]. The disease affects both sexes. CML is most commonly diagnosed between 40 and 70 years of age, with the peak being recorded around the age of 55. The survival rate is estimated at 96% at 1 year and 85% at 5 years [8].

The introduction of tyrosine kinase inhibitors (TKIs) has led to a revolution in the treatment of CML. Imatinib was the first BCR::ABL1 tyrosine kinase inhibitor approved for CML and remains one of several first-line treatment options for patients with newly diagnosed chronic-phase CML [9,10]. Imatinib specifically blocks ATP binding to the ABL fragment of the oncogene BCR::ABL1 to suppress tumor progression and prevent autophosphorylation and downstream signaling, leading to growth arrest and apoptosis.

In cases of resistance or intolerance to imatinib, second- and third-generation TKIs are available. Second-generation TKIs such as nilotinib and dasatinib have relatively high affinities for the BCR::ABL1 kinase domain and are commonly used in patients who fail first-line therapy. Third-generation inhibitors, including ponatinib, which is effective against the T315I mutation, and the allosteric inhibitor asciminib, have further expanded therapeutic options, enabling targeted management of resistant or refractory CML patients. These advances have markedly improved long-term outcomes and deep molecular responses in CML patients [11,12].

Despite the effectiveness of imatinib, TKI resistance remains a major clinical challenge [13]. According to the literature, well-established resistance mechanisms include changes in the BCR::ABL1 kinase domain, activation of various signaling pathways, changes in the microenvironment, overexpression of drug efflux pumps and epigenetic modifications [14,15]. Recent studies have shown that resistance to imatinib can also be linked to a complex metabolic adaptation for the survival and proliferation of resistant leukemia cells [16].

The metabolism of cancer cells is markedly disrupted. They rely on specific metabolic pathways for their survival and accelerated growth, including glycolysis, fatty acid oxidation, and the pentose phosphate pathway [17]. As described by Otto Warburg in 1956, cancer cells preferentially utilize aerobic glycolysis rather than oxidative phosphorylation (OXPHOS). However, leukemic stem cells diverge from this pattern. They rely more on oxidative phosphorylation [18]. Whether this metabolic profile differs according to imatinib sensitivity and resistance status in CML cells remains incompletely understood, and characterizing the intrinsic metabolic phenotype of sensitive and resistant CML cell lines may help identify metabolic features associated with the resistant state [19,20].

This metabolic transition to OXPHOS has been associated with functional changes in mitochondria, notably an increase in the production of reactive oxygen species (ROS) [21]. Higher levels of ROS lead to oxidative damage and disrupt normal metabolism, inducing cellular stress and apoptosis. In response to oxidative stress, CML cells activate adaptive responses, such as autophagy [22,23].

Autophagy is a conserved intracellular recycling process that maintains cellular homeostasis by degrading damaged organelles and misfolded proteins. In CML, autophagy plays a dual role: it limits excessive ROS accumulation through mitophagy while also serving as a survival pathway enabling leukemic cells to cope with metabolic and oxidative stress. The principal marker of autophagosome formation is microtubule-associated protein 1 light chain 3 (LC3), which exists in two forms: cytosolic LC3-I and autophagosome-associated LC3-II. The conversion of LC3-I to LC3-II reflects autophagosome formation and is widely used as an indicator of autophagic activity. p62 (sequestosome-1) is a selective autophagy receptor that binds ubiquitinated cargo and is itself degraded upon autophagosome-lysosome fusion. Although low p62 levels could be indicative of increased autophagic degradation, p62 levels alone cannot confirm autophagic flux, whereas p62 accumulation is indicative of impaired or defective autophagy [24].

To investigate metabolic and autophagy-related alterations associated with imatinib resistance, we used a K562-derived cellular model comprising parental K562 cells, an imatinib-sensitive clone (K562-S), and its imatinib-resistant counterpart (K562-R), which was originally characterized as resistant to 1 µM imatinib, with the main mechanism underlying resistance in these cells principally attributed to the compromised autophosphorylation of BCR::ABL1 [25]. The use of these three related cell lines enables a comparative evaluation across different sensitivity stages, from the baseline parental phenotype (K562) to high sensitivity (K562-S) and established resistance (K562-R).

This study aimed to characterize the basal metabolic profile (glycolytic and mitochondrial respiratory functions) of K562, K562-S, and K562-R cells and to assess their levels of ROS and NO and autophagy markers (LC3 and p62) in response to imatinib treatment at each cell line’s respective IC_50_ concentration.

## 2. Materials and Methods

### 2.1. Cell Culture and Reagents

The human chronic myeloid leukemia cell lines K-562 (ATCC^®^ CCL-243™), imatinib-sensitive K562-S (ATCC^®^ CRL-3343™), and imatinib-resistant K562-R (ATCC^®^ CRL-3344™) were obtained from the American Type Culture Collection (ATCC; Manassas, VA, USA). For consistency, the cell lines are hereafter referred to as K562, K562-S, and K562-R, respectively. The cells were grown in suspension in RPMI-1640 medium (ATCC^®^ 30-2001™) supplemented with 10% heat-inactivated fetal bovine serum (ATCC^®^ 30-2020™), 2 mM L-glutamine (ATCC^®^ 30-2214™), and 1% penicillin–streptomycin (Gibco, Thermo Fisher Scientific, Waltham, MA, USA; cat. no. 15140-122). Cultures were maintained at 37 °C in a humidified atmosphere containing 5% CO_2_. To maintain the resistant phenotype, K562-R cells were routinely cultured in complete RPMI-1640 medium supplemented with 1 µM imatinib, which was freshly added at each reseeding. Imatinib (Zenith Pharma, Casablanca, Morocco) was prepared as a 100 mM stock solution in dimethyl sulfoxide (DMSO; Thermo Scientific Acros, Geel, Belgium; cat. no. D1391), aliquoted, and stored at −20 °C until use. Before the experimental assays, which included metabolic activity, the quantification of cellular reactive oxygen species and nitric oxide levels, and the quantification of autophagy markers, the K562-R cells were washed and cultured for 48 h in imatinib-free complete medium. This 48 h washout period was applied to both the imatinib-treated and vehicle-treated K562-R cells. The experimental vehicle controls received an equivalent volume of DMSO, and the final DMSO concentration did not exceed 0.1% (*v*/*v*).

### 2.2. MTT Assay

The effect of imatinib on the metabolic activity of K562, K562-S, and K562-R cells was evaluated via the 3-(4,5-dimethylthiazol-2-yl)-2,5-diphenyltetrazolium bromide (MTT) assay (Promega, Madison, WI, USA) as previously described [26]. Before the experiment, K562-R cells routinely maintained in medium containing 1 µM imatinib were washed and cultured for 48 h in imatinib-free complete medium. The cells were counted via trypan blue exclusion and seeded in 96-well plates at a density of 5 × 10^4^ viable cells per well in 100 µL of complete RPMI-1640 medium.

The cells were exposed to imatinib at final concentrations of 0.1, 0.25, 0.50, and 1 µM for 72 h at 37 °C in a humidified atmosphere containing 5% CO_2_. The control cells received an equivalent volume of vehicle, and the final DMSO concentration was maintained at ≤0.1% (*v*/*v*) in all the wells. Wells containing culture medium without cells were included as blanks.

Following treatment, MTT solution was added to each well to obtain a final concentration of 0.5 mg/mL, and the plates were incubated for an additional 2 h at 37 °C in the dark. Because K562 cells grow in suspension, the plates were centrifuged to pellet the cells, and the supernatant was carefully removed without disturbing the cell pellet. The resulting formazan crystals were dissolved in 100 µL of DMSO, and the absorbance was measured at 570 nm via a FlexStation microplate reader (Molecular Devices, Sunnyvale, CA, USA).

After subtraction of the corresponding blank absorbance, metabolic activity was calculated according to the following equation:

$$Relative\;metabolic\;activity\left(\%\right)=\frac{Absorbance\;of\;treated\;cells-Absorbance\;of\;blank\;cells}{Absorbance\;of\;vehicle\;cells-Absorbance\;of\;blank\;cells}\times 100$$

Vehicle-treated cells were defined as those with 100% metabolic activity.

Dose-response curves were generated by plotting the relative metabolic activity against the imatinib concentration for each cell line, and the half-maximal inhibitory concentration (IC_50_) was determined via nonlinear regression in GraphPad Prism 10. For K562-R cells, the IC_50_ was not within the tested range; therefore, 1 µM, the highest concentration tested, was used for downstream experiments.

### 2.3. Metabolic Analysis

The metabolic profiles of K562, K562-S, and K562-R cells were assessed by measuring the oxygen consumption rate (OCR), an indicator of mitochondrial respiration, and the extracellular acidification rate (ECAR), an indicator of glycolytic activity, via a Seahorse XF24 Extracellular Flux Analyzer (Agilent, Billerica, MA, USA) according to the manufacturer’s instructions [27].

The day before each assay, the sensor cartridge was hydrated overnight in calibration buffer supplied by the manufacturer. Seahorse XF24 microplate wells were coated with 25 µL of Cell-Tak solution (Corning, NY, USA. cat. 354240; 22.4 µg/mL) and incubated at 4 °C overnight to facilitate cell adhesion. The cells were seeded at a density of 2 × 10^5^ cells/well and allowed to rest for 1 h at 37 °C in a non-CO_2_ humidified incubator in Seahorse XF DMEM Base Medium (pH 7.4, cat. 103575-100; Agilent, Billerica, MA, USA) supplemented with 5.6 mM glucose (Agilent Technologies, Santa Clara, CA, USA; cat. no. 103577-100), 2 mM L-glutamine, and 1 mM sodium pyruvate (Agilent Technologies; cat. no. 103578-100) without sodium bicarbonate or phenol red.

These assays were performed under basal conditions without imatinib or vehicle treatment to characterize the intrinsic metabolic phenotype of each cell line.

### 2.4. Glycolysis Stress Test

The Glycolysis Stress Test was used to assess key parameters of glycolytic flux. Non-glycolytic acidification represents extracellular acidification arising from sources independent of glycolysis and is measured prior to glucose injection. Glycolysis is measured as the ECAR level reached after the injection of saturating amounts of glucose. Glycolytic capacity, determined as the maximum ECAR observed following oligomycin addition, reflects how effectively a cell can upregulate glycolysis when oxidative phosphorylation is inhibited. The glycolytic reserve, calculated as the difference between glycolytic capacity and basal glycolysis, is a measure of a cell’s ability to respond to increased energy demands under stress conditions.

Prior to the assay, the culture medium was replaced with Seahorse XF DMEM Base Medium (pH 7.4; Agilent, Billerica, MA, USA) without glucose or pyruvate to allow measurement of non-glycolytic acidification at baseline. Sequential injections were then performed with glucose (10 mM) (Agilent Technologies, Santa Clara, CA, USA) as an energetic substrate, oligomycin (Agilent Technologies) (1 µM; ATP synthase inhibitor) to drive maximal glycolytic capacity, and 2-deoxyglucose (2-DG Agilent Technologies) (50 mM) to competitively inhibit glycolysis and confirm ECAR specificity. Glycolytic parameters were calculated according to the manufacturer’s recommendations [27].

### 2.5. Mitochondrial Stress Test

The Seahorse XF Cell Mito Stress Test directly measures the OCR to evaluate mitochondrial function. Basal respiration reflects the energy used to meet the cell’s endogenous ATP demand and drive proton leakage pathways, whereas ATP-linked respiration quantifies respiration coupled with mitochondrial ATP synthesis, which is sensitive to oligomycin. Proton leakage, which occurs independently of ATP synthase, regulates the membrane potential and can serve as a cytoprotective mechanism. Maximal respiration, assessed following injection of the protonophore carbonyl cyanide-p-trifluoromethoxy-phenylhydrazone (FCCP), represents the peak respiratory rate achieved upon full uncoupling of the inner mitochondrial membrane. The spare respiratory capacity, calculated as the difference between maximal and basal respiration, reflects a cell’s ability to respond to increased energy demands under stress conditions. Nonmitochondrial respiration is determined following sequential injection of rotenone (a complex I inhibitor) and antimycin A (a complex III inhibitor), which together abolish mitochondrial electron transport chain activity.

The cells were subsequently resuspended in Seahorse XF DMEM base medium (pH 7.4; Agilent, Santa Clara, CA, USA) supplemented with 5.6 mM glucose, 2 mM L-glutamine, and 1 mM sodium pyruvate. Mitochondrial function was assessed by sequential injections of oligomycin (1 µM), FCCP (1 µM), and rotenone/antimycin A (0.5 µM each), all obtained from Agilent Technologies (Santa Clara, CA, USA). The mitochondrial respiratory parameters were calculated according to the manufacturer’s recommendations.

### 2.6. Quantification of Cellular Reactive Oxygen Species

Intracellular reactive oxygen species (ROS) levels were assessed using the Cellular Reactive Oxygen Species Detection Assay Kit (Red Fluorescence) (No. ab186027; Abcam, Cambridge, UK) in K562, K562-S, and K562-R cells. This fluorometric assay employs a cell-permeable redox-sensitive probe that is oxidized by intracellular ROS, including superoxide and hydroxyl radicals, generating a red fluorescent signal proportional to the overall intracellular ROS level. Each cell line was treated with imatinib at its corresponding IC_50_ concentration, as determined by the MTT assay (0.445 μM for K562, 0.137 μM for K562-S, and 1 μM for K562-R, the highest tested concentration used for this cell line as described in Section 2.2), for 72 h. Corresponding vehicle-treated cells were included as controls. Cells were then seeded into a 96-well plate at a density of 2 × 10^4^ cells per well and incubated with the ROS Red working solution, prepared according to the manufacturer’s instructions, for 60 min at 37 °C. The fluorescence signal was analyzed at Ex/Em = 520/605 nm via a FlexStation II microplate reader (Molecular Devices, Sunnyvale, CA, USA).

### 2.7. Quantification of Nitric Oxide Levels

Nitric oxide (NO) production was indirectly assessed by quantifying the total nitrate/nitrite concentration accumulated in the culture medium using the Nitric Oxide Assay Kit (Fluorometric) (No. ab65327; Abcam, Cambridge, UK). Since NO is rapidly converted into nitrite (NO_2_^−^) and nitrate (NO_3_^−^), the total nitrate/nitrite concentration was used as an indicator of NO production. The assay is based on the enzymatic conversion of nitrate to nitrite by nitrate reductase, followed by the reaction of nitrite with the fluorescent probe 2,3-diaminonaphthalene (DAN), generating a fluorescent product proportional to the total NO production. A cell suspension was prepared by seeding K562, K562-S, and K562-R cells in 6-well plates at a density of 1 × 10^6^ cells/well in 2 mL of culture medium. Cells were treated with vehicle or imatinib at the corresponding IC_50_ concentration for each cell line (0.445 μM for K562, 0.137 μM for K562-S, and 1 μM for K562-R, as determined by the MTT assay described in Section 2.2) for 72 h. Culture supernatants were then collected, and NO production was determined according to the manufacturer’s instructions. The fluorescence levels of the reaction mixtures were measured at Ex/Em = 360/450 nm via a FlexStation II microplate reader (Molecular Devices, Sunnyvale, CA, USA).

### 2.8. Western Blot Analysis

K562, K562-S, and K562-R cells were treated for 72 h with vehicle or imatinib at concentrations corresponding to their respective 72-h MTT-derived IC_50_ values: 0.445 µM for K562, 0.137 µM for K562-S, and 1 µM for K562-R cells. Following treatment, the cells were lysed in radioimmunoprecipitation assay Pierce RIPA Buffer (Thermo Fisher Scientific, Rockford, IL, USA) supplemented with a protease inhibitor cocktail (200×; Cell Signaling Technology, Danvers, MA, USA; cat. no. 7012). Total protein concentrations were determined via a BCA protein assay kit (Thermo Fisher Scientific, Rockford, IL, USA). Proteins were separated on 10% SDS-PAGE gels and subsequently transferred onto polyvinylidene fluoride (PVDF) membranes. To block nonspecific binding, the membranes were incubated in 5% nonfat dry milk for 2 h at room temperature and then washed three times for 10 min each with PBS containing 0.1% Tween-20 (PBST). The membranes were incubated overnight at 4 °C with rabbit polyclonal anti-p62 antibody (Abcam, Cambridge, UK; ab91526; 1:1000), rabbit polyclonal anti-LC3B antibody (Abcam, ab51520; 1:1000), and mouse monoclonal anti-GAPDH antibody (Abcam, ab8245; 1:1000). After three additional 10 min washes with PBST, the membranes were incubated for 1 h at room temperature with fluorescent secondary antibodies (goat anti-rabbit and goat anti-mouse, Abcam; 1:5000). The membranes were then washed three times for 10 min each with PBST. The protein bands were visualized via the LI-COR Odyssey imaging system (LI-COR, Inc., Lincoln, NE, USA). Band intensities were quantified via Image Studio software, version 6.0, and normalized to the level of GAPDH, which was used as the loading control. Each condition was assessed in six independent experiments (*n* = 6).

### 2.9. Statistical Analysis

Statistical analyses were performed via GraphPad Prism 10. Data normality was assessed via the Shapiro-Wilk test. Two-way ANOVA was used to analyze the effects of the cell line and imatinib concentration on metabolic activity (MTT assay) and the effects of the cell line and treatment on the ROS levels, NO levels, and autophagy-related markers (p62, LC3-I, LC3-II, and the LC3-II/LC3-I ratio). One-way ANOVA was used to assess differences in metabolic stress test parameters (glycolytic and mitochondrial function) among the three cell lines. Bonferroni post hoc test was used for multiple comparisons. The results are expressed as the mean ± standard error of the mean (SEM), and statistical significance was set at *p* < 0.05.

## 3. Results

### 3.1. Differential Effects of Imatinib on the Metabolic Activity of the K562, K562-S, and K562-R Cell Lines

To evaluate the sensitivity of CML cell lines to imatinib, metabolic activity was assessed via an MTT assay in parental K562, imatinib-sensitive K562-S, and imatinib-resistant K562-R cells treated with increasing concentrations of imatinib (0–1 µM) for 72 h. Two-way ANOVA revealed a significant interaction between the imatinib concentration and the cell line (F (8,75) = 11.68, *p* < 0.0001), indicating that the metabolic response to imatinib differed significantly among the three CML cell lines. Significant main effects were also observed for the imatinib concentration (F (4,75) = 88.28, *p* < 0.0001) and cell line (F (2,75) = 114.3, *p* < 0.0001). Bonferroni post hoc comparisons revealed that, at 0.10 µM imatinib, K562-S cells exhibited significantly lower metabolic activity than both K562 and K562-R cells (both *p* < 0.0001), whereas no significant difference was observed between K562 and K562-R cells. At 0.25, 0.50, and 1.00 µM, K562-S cells presented lower metabolic activity than parental K562 cells (*p* < 0.05, *p* < 0.05, and *p* < 0.01, respectively), whereas K562-R cells maintained significantly greater metabolic activity than both K562 and K562-S cells at each of these concentrations (all *p* < 0.0001) (Figure 1).

IC_50_ values were estimated via nonlinear regression using an inhibitor-versus-normalized-response model with a variable slope in GraphPad Prism version 10. K562-S cells exhibited the highest sensitivity to imatinib, with an estimated IC_50_ of 0.137 µM (95% CI: 0.076–0.196 µM), whereas parental K562 cells showed intermediate sensitivity, with an estimated IC_50_ of 0.445 µM (95% CI: 0.352–0.579 µM). In contrast, K562-R cells retained more than 75% of their relative metabolic activity at the highest tested concentration. Consequently, the IC_50_ was not reached within the 0.10–1.00 µM concentration range and was considered to be greater than 1 µM under the present experimental conditions.

### 3.2. Compared with the Parental Line, the Imatinib-Sensitive and Imatinib-Resistant K562 Cell Lines Exhibit Reduced Glycolytic Activity

Glycolytic function was assessed via the Seahorse XF glycolytic stress test (Figure 2a). One-way ANOVA revealed a significant effect on glycolysis (F (2,15) = 15.63, *p* < 0.001), with both K562-S and K562-R cells showing significantly reduced glycolytic activity compared with parental K562 cells (*** *p* < 0.001 for both). No significant difference was observed between K562-S and K562-R cells (Figure 2b). Glycolytic capacity was also significantly affected (F (2,15) = 19.11, *p* < 0.0001), with reductions in both K562-S (* *p* = 0.0190) and K562-R (**** *p* < 0.0001) cells compared with K562 cells and a further significant reduction in K562-R cells compared with K562-S cells (* *p* = 0.0264) (Figure 2c). Non-glycolytic acidification had a significant effect on the cell line (F (2,15) = 5.039, *p* = 0.0212); however, only K562-R cells differed significantly from parental K562 cells (* *p* = 0.0246), while the difference in K562-S cells did not reach significance (*p* = 0.1084) (Figure 2d). No significant differences were detected in the glycolytic reserve (F (2,15) = 2.994, *p* = 0.0805) or the glycolytic reserve% (F (2,15) = 1.027, *p* = 0.3820) across the three cell lines (Figure 2e,f).

### 3.3. Mitochondrial Function Is Altered in Imatinib-Sensitive and Imatinib-Resistant K562 Cells

Mitochondrial function was assessed via the Seahorse XF mitochondrial stress test in parental K562, K562-S, and K562-R cells (Figure 3a). No significant differences were observed in ATP-linked respiration (F (2,15) = 3.354, *p* = 0.0625) or mitochondrial coupling efficiency (F (2,15) = 0.9731, *p* = 0.4005) across the three cell lines (Figure 3b,c). Compared with that in parental K562 cells, maximal respiration was significantly lower in both K562-S and K562-R cells (F (2,15) = 81.57, *p* < 0.0001; **** *p* < 0.0001 for both comparisons), indicating a global reduction in the mitochondrial respiratory reserve in both sublines (Figure 3d). Basal respiration differed significantly among the three cell lines (F (2, 15) = 6.158, *p* = 0.0112), with K562-S cells showing significantly greater basal OCRs than K562-R cells (# *p* = 0.0109) (Figure 3e). Proton leakage was significantly lower in K562-R cells than in parental K562 cells (F (2,15) = 4.231, *p* = 0.0349; * *p* = 0.0370), suggesting reduced mitochondrial uncoupling in resistant cells (Figure 3f). The spare respiratory capacity significantly differed among the cell lines (F (2,15) = 19.11, *p* < 0.001). This parameter was significantly lower in K562-S cells than in both K562 (*** *p* < 0.001) and K562-R cells (## *p* = 0.004), whereas K562-R cells maintained a relatively preserved spare capacity (Figure 3g). A similar pattern was observed for spare respiratory capacity% (F (2,15) = 9.441, *p* = 0.0022), with K562-S cells showing significantly lower values than both K562 cells (* *p* = 0.0141) and K562-R cells (** *p* = 0.0029) (Figure 3h). Collectively, these data indicate that, compared with K562-S cells, K562-R cells exhibit a distinct mitochondrial phenotype characterized by reduced basal respiration and proton leakage but relatively preserved spare respiratory capacity. This profile indicates the maintenance of respiratory reserve despite lower basal mitochondrial activity, rather than a global upregulation of OXPHOS.

### 3.4. Imatinib Differentially Increases the ROS and NO Levels Across CML Cell Sublines

To assess whether imatinib treatment induces oxidative stress and alters NO production in a manner dependent on the resistance phenotype, intracellular ROS levels and NO production were measured in K562, K562-S, and K562-R cells treated for 72 h with imatinib at the MTT-derived IC_50_ concentrations for K562 and K562-S cells (0.445 and 0.137 µM, respectively), and at 1 µM for K562-R cells, the highest concentration tested, or with vehicle. Because the three cell lines were exposed to different imatinib concentrations, statistical comparisons of treatment effects were restricted to vehicle versus imatinib within each cell line rather than direct comparisons between cell lines. Two-way ANOVA revealed a significant interaction effect between treatment and cell line on the ROS level (F (2,30) = 6.033, *p* = 0.0063), indicating that the magnitude of the imatinib-induced change in the ROS level differed among the three cell lines. Significant main effects of cell line (F (2,30) = 6.264, *p* = 0.0053) and treatment (F (1,30) = 29.23, *p* < 0.0001) were also observed. Bonferroni post hoc comparisons revealed that, compared with vehicle, imatinib significantly increased ROS levels in K562 (*p* < 0.0001) and K562-S cells (*p* = 0.0080), whereas no significant change was detected in K562-R cells treated with 1 µM imatinib cells (*p* > 0.9999) (Figure 4a). Because the three cell lines were exposed to different imatinib concentrations, these within-cell-line responses should not be interpreted as direct quantitative evidence of differential oxidative-stress susceptibility between the cell lines.

In contrast, two-way ANOVA revealed no significant interaction between treatment and cell line (F (2,30) = 2.770, *p* = 0.0787), indicating that the magnitude of the imatinib-induced increase in NO was relatively consistent across the three cell lines. Significant main effects of cell line (F (2,30) = 3.867, *p* = 0.0320) and treatment (F (1,30) = 94.34, *p* < 0.0001) were observed. Bonferroni post hoc comparisons confirmed that imatinib significantly increased NO levels relative to the vehicle in K562 (*p* < 0.0001), K562-S (*p* < 0.0001), and K562-R cells (*p* = 0.0006) (Figure 4b). Imatinib treatment significantly increased total nitrate/nitrite levels in each of the three cell lines compared with their respective vehicle controls. Collectively, these findings indicate that the response to imatinib differed between the two markers. Whereas ROS accumulation was restricted to K562 and K562-S cells, NO production increased consistently across all three CML cell lines. The absence of increased ROS in K562-R cells despite increased NO levels suggests that resistant cells may activate antioxidant mechanisms that limit ROS accumulation while preserving NO responsiveness.

### 3.5. Imatinib Differentially Modulates Autophagy Markers in K562, K562-S, and K562-R Cells

To determine whether imatinib modulates autophagic markers in a manner dependent on the resistance phenotype, the levels of p62 and LC3 (LC3-I, LC3-II, and the LC3-II/LC3-I ratio) were assessed by Western blot in K562, K562-S, and K562-R cells treated for 72 h with imatinib (0.445 µM for K562, 0.137 µM for K562-S, and 1 µM for K562-R) or with vehicle. For the ROS and NO assays, statistical comparisons were restricted to vehicle versus imatinib within each cell line.

For p62, two-way ANOVA revealed no significant interaction effect between treatment and cell line (F (2,30) = 2.776, *p* = 0.0783), whereas a significant main effect of treatment was observed (F (1,30) = 7.560, *p* = 0.0100). Bonferroni post hoc comparisons revealed that imatinib significantly decreased p62 levels in K562-S cells (*p* = 0.0215), whereas no significant change was observed in K562 (*p* = 0.1149) or K562-R cells (*p* > 0.9999) (Figure 5a).

Similarly, for LC3-I, two-way ANOVA revealed no significant interaction effect (F (2,30) = 2.594, *p* = 0.0914), with a significant main effect of treatment (F (1,30) = 5.426, *p* = 0.0268). Imatinib significantly decreased LC3-I levels in K562 cells (*p* = 0.0179), with no significant change in K562-S (*p* = 0.5722) or K562-R cells (*p* > 0.9999) (Figure 5b). In contrast, the LC3-II levels were not affected by treatment in any cell line (interaction: F (2,30) = 0.1107, *p* = 0.8956; treatment: F (1,30) = 1.148, *p* = 0.2924), although a significant main effect of cell line was observed (F (2,30) = 6.462, *p* = 0.0046), reflecting differences in the basal LC3-II levels among the three lines (Figure 5c).

Analysis of the LC3-II/LC3-I ratio revealed a significant interaction effect between treatment and cell line (F (2,30) = 4.412, *p* = 0.0209), together with significant main effects of cell line (F (2,30) = 7.816, *p* = 0.0019) and treatment (F (1,30) = 7.658, *p* = 0.0096). Bonferroni post hoc comparisons revealed that imatinib markedly increased the LC3-II/LC3-I ratio in K562 cells (*p* = 0.0014), whereas no significant change was observed in K562-S (*p* = 0.9889) or K562-R cells (*p* > 0.9999) (Figure 5d).

Collectively, these findings indicate that imatinib alters the steady-state levels of autophagy-related markers in a cell-line-dependent manner. In parental K562 cells, the decrease in LC3-I accompanied by an increased LC3-II/LC3-I ratio indicates an altered LC3 profile following imatinib treatment; however, these steady-state measurements do not establish changes in autophagic flux. In K562-S cells, the decrease in p62 was not accompanied by a significant change in the LC3-II/LC3-I ratio. Therefore, this isolated change in p62 cannot be interpreted as evidence of altered autophagic flux. In K562-R cells, none of the autophagy-related markers examined were significantly altered by imatinib under the experimental conditions tested.

## 4. Discussion

The objective of this study was to investigate metabolic alterations associated with imatinib sensitivity and resistance in CML cell lines, with a particular focus on the interplay between energy metabolism, oxidative stress, and autophagy. By comparing parental K562, imatinib-sensitive K562-S, and imatinib-resistant K562-R cells, we characterized distinct metabolic, redox, and autophagy-related features associated with different imatinib sensitivity profiles.

### 4.1. Imatinib Differentially Affects Metabolic Activity Across CML Cell Sublines

Following 72 h of imatinib treatment, metabolic activity decreased in both K562-S and parental K562 cells, with estimated IC_50_ values of 0.137 µM (95% CI: 0.076–0.196 µM) and 0.445 µM (95% CI: 0.352–0.579 µM), respectively, as determined by nonlinear regression. In contrast, K562-R cells maintained high metabolic activity throughout the concentration range tested, and a reliable IC_50_ could not be estimated within this range, which is consistent with a markedly resistant phenotype. This differential response is consistent with previous reports showing that imatinib-sensitive CML cells undergo pronounced metabolic suppression following BCR::ABL1 inhibition, whereas resistant cells preserve their metabolic activity and display reduced susceptibility to the drug [28]. Similarly, Yalda et al. reported that imatinib-resistant K562 sublines (K562r0.6 and K562r5) retained substantially greater cell viability, as assessed by the MTT assay, than their sensitive counterparts at equivalent concentrations, although a dose-dependent reduction was still observed at higher concentrations (up to 5 µM), suggesting that resistance reflects a shift in the sensitivity threshold rather than a complete loss of drug responsiveness [29]. Together, these findings support an association between imatinib resistance and altered metabolic characteristics in CML cells.

### 4.2. Glycolytic Activity Is Reduced in Both Sensitive and Resistant Sublines

Compared with parental K562 cells, both K562-S and K562-R cells presented significantly reduced glycolysis and glycolytic capacity, with no significant difference in basal glycolysis between the two sublines. However, the glycolytic capacity was significantly lower in K562-R cells than in K562-S cells, indicating a further reduction in maximal glycolytic capacity in the resistant subline. These findings are consistent with the work of De Rosa et al., who demonstrated that imatinib modulates the expression of glycolytic enzymes, including hexokinase II, phosphorylated PKM2, and lactate dehydrogenase A, as well as mitochondrial complex subunits, thereby altering glucose metabolism [20]. The comparable reduction in basal glycolysis observed in K562-S and K562-R cells suggests that basal glycolytic activity alone does not distinguish the sensitive and resistant sublines, whereas their mitochondrial respiratory profiles differed.

### 4.3. K562-S and K562-R Cells Exhibit Distinct Mitochondrial Phenotypes

Compared with K562-R cells, K562-S cells presented increased basal respiration, whereas K562-R cells exhibited reduced basal respiration, reduced proton leakage, and relatively preserved spare respiratory capacity. These findings do not support a uniform upregulation of OXPHOS in resistant cells; rather, they identify a distinct mitochondrial respiratory phenotype characterized by lower basal activity while retaining respiratory reserve. Although preserved spare respiratory capacity may reflect an ability to accommodate increased energetic demand, the present study did not functionally determine whether this respiratory reserve contributes to imatinib resistance. Establishing such a relationship would require metabolic perturbation experiments, for example through pharmacological inhibition of mitochondrial respiration or glycolysis followed by assessment of imatinib sensitivity. These observations are nonetheless consistent with previous reports showing that imatinib-resistant CML stem and progenitor cells upregulate TCA cycle enzymes and exhibit increased oxygen consumption under specific conditions [18,30], suggesting that the relationship between resistance and mitochondrial activity may be context dependent.

### 4.4. Oxidative Stress Is Differentially Regulated in Sensitive and Resistant Cells

Imatinib significantly increased ROS levels in K562 and K562-S cells, whereas no significant ROS increase was detected in K562-R cells treated with 1 µM imatinib. This differential pattern is of interest; however, it should be interpreted cautiously because the three cell lines were exposed to different imatinib concentrations according to their sensitivity profiles, and treatment responses were therefore evaluated only within each cell line. Consequently, the absence of a significant ROS increase in K562-R cells cannot be interpreted as direct evidence that resistant cells are intrinsically protected from imatinib-induced oxidative stress. Whether antioxidant or other redox-regulatory mechanisms contribute to the observed response in K562-R cells remains to be determined experimentally. In contrast to the ROS response, total nitrate/nitrite levels increased significantly following imatinib treatment within each of the three cell lines. Because different imatinib concentrations were used and cross-cell-line treatment responses were not directly compared, these findings do not establish whether the magnitude of the NO-related response differs according to imatinib sensitivity. The relationship between ROS and NO is redox sensitive: elevated ROS have been reported to stimulate NO production in several cellular models [31,32]. These observations are supported by a clinical study in patients with CML in Tunisia, in which imatinib-resistant patients displayed a disrupted oxidative profile compared with their sensitive counterparts [21]. However, the mechanistic basis of antioxidant adaptation in K562-R cells was not directly investigated in the present study, and further experiments targeting specific antioxidant pathways, such as NRF2 or glutathione metabolism, are needed to confirm this interpretation.

### 4.5. Autophagy-Related Alterations Differ Between Sensitive and Resistant CML Cell Lines

Imatinib treatment altered autophagy-related markers in K562 and K562-S cells, although the patterns differed between the two cell lines. K562-S cells exhibited decreased p62 levels without significant changes in LC3-I, LC3-II, or the LC3-II/LC3-I ratio. Because p62 levels can be influenced by processes other than autophagic degradation, and because autophagic flux was not directly assessed, this isolated decrease cannot be interpreted as evidence of enhanced autophagy. In parental K562 cells, imatinib decreased LC3-I and increased the LC3-II/LC3-I ratio, whereas LC3-II levels remained unchanged. These findings indicate an alteration in the steady-state LC3 profile following imatinib exposure but, in the absence of lysosomal inhibition experiments, do not establish increased autophagic flux. In K562-R cells, none of the autophagy-related markers examined were significantly altered by imatinib under the experimental conditions tested. These findings are consistent with reports showing that imatinib induces autophagy in a dose-dependent manner in K562 cells, with the upregulation of ATG5 and Beclin-1 [33]. These changes in autophagy-related markers can be considered in the context of previous studies reporting imatinib-induced autophagy in K562 cells, alongside apoptosis and senescence and that inhibition of ATG7 or Beclin-1 reduced the senescent response, suggesting that autophagy contributes to the adaptation of K562 cells to imatinib exposure [34].

In contrast, the absence of significant changes in the autophagy-related markers examined in K562-R cells differs from reports describing enhanced basal autophagic activity in imatinib-resistant K562 sublines, characterized by lower basal p62 and a higher LC3-II/LC3-I ratio than in parental cells, as well as accelerated LC3-II accumulation and p62 degradation following high-dose imatinib exposure (5 µM) [35]. Similarly, resistant K562 cells reportedly display increased autophagy activation associated with increased GCA expression, which acts through the TRAF6–ULK1 axis to promote resistance [36]. These discrepancies may be attributable to differences in experimental design: whereas the present study assessed autophagy markers exclusively in relation to each cell line’s own IC_50_-equivalent imatinib concentration, the cited studies either compared basal autophagy between resistant and parental lines independently of treatment or examined responses to substantially higher imatinib concentrations (5 µM) than those used here (1 µM for K562-R) [37,38]. These discrepancies may reflect differences in cell models, imatinib concentrations, treatment duration, and experimental approaches used to assess autophagy. Importantly, because the present study did not directly measure autophagic flux, the absence of significant changes in p62 and LC3 markers in K562-R cells should not be interpreted as evidence of absent autophagic activity.

Beyond targeting metabolic and stress-adaptation pathways, emerging therapeutic approaches seek to overcome TKI resistance by directly reducing BCR::ABL1 protein abundance rather than solely inhibiting its kinase activity. Recent work by Yang et al. demonstrated in Philadelphia chromosome-positive acute lymphoblastic leukemia models that heat stress promoted ubiquitin–proteasome-mediated degradation of BCR::ABL1, including drug-resistant variants, and impaired the growth of BCR::ABL1-driven leukemia cells [39]. Although these findings were obtained in Ph+ ALL rather than CML models, they highlight BCR::ABL1 protein stability as a potentially relevant therapeutic axis for BCR::ABL1-dependent drug resistance. Such approaches provide a complementary perspective to the metabolic and stress-response adaptations investigated here and illustrate that strategies targeting both the oncogenic driver and adaptive cellular programs may warrant further investigation in resistant leukemia.

### 4.6. Limitations and Perspectives

The present study has several limitations that should be acknowledged. All the experiments were conducted exclusively with K562-derived sublines, which represent a single genetic background and do not fully recapitulate the complexity of the bone marrow microenvironment or the heterogeneity of primary CML cells. Validation of these findings in additional CML cell lines, such as KCL-22 or LAMA84, or in primary CD34+ cells from CML patients, would substantially strengthen the generalizability of our conclusions and is planned as a priority in future work. In addition, the metabolic analyses performed in this study were descriptive and were designed to characterize the basal glycolytic and mitochondrial phenotypes of the three cell lines. Although K562-R cells exhibited a distinct mitochondrial profile with relatively preserved spare respiratory capacity, the functional contribution of this phenotype to imatinib resistance was not directly tested. Pharmacological or genetic perturbation of mitochondrial respiration and glycolysis, combined with assessment of imatinib sensitivity, will therefore be required to determine whether these metabolic features contribute causally to the resistant phenotype. Furthermore, this study focused solely on imatinib; whether similar metabolic adaptations occur in response to second- or third-generation TKIs such as dasatinib or ponatinib remains to be determined. With respect to autophagy, the present study relied on steady-state levels of p62 and LC3-I/LC3-II, which reflect a snapshot of autophagy-related markers rather than a dynamic measure of autophagic flux. The use of lysosomal inhibitors such as bafilomycin A1 or chloroquine is required to distinguish changes in autophagosome formation from altered autophagosome clearance and thereby establish whether autophagic flux is increased, decreased, or unchanged. Additionally, the K562-R cell line used in this study exhibited a markedly resistant phenotype for which a reliable IC_50_ could not be established within the tested concentration range (0.1–1 μM); consequently, the highest tested concentration was used as a pragmatic treatment dose for downstream experiments. This approach, while necessary given the experimental constraints, may not fully capture the concentration-dependent responses that could emerge at higher imatinib concentrations, as suggested by studies reporting continued dose-dependent effects in resistant K562 sublines at concentrations up to 5 µM.

## 5. Conclusions

In conclusion, this study identifies distinct metabolic and stress-response profiles among K562-derived cell lines with different sensitivities to imatinib. Both K562-S and K562-R cells exhibited reduced glycolytic activity compared with parental K562 cells, while K562-R cells displayed a distinct mitochondrial phenotype characterized by lower basal respiration than K562-S cells and relatively preserved spare respiratory capacity. Following imatinib treatment, ROS levels increased in K562 and K562-S cells but not in K562-R cells under the respective treatment conditions, whereas total nitrate/nitrite levels increased in all three cell lines. Imatinib also differentially altered steady-state autophagy-related markers, with changes in the LC3 profile in parental K562 cells, decreased p62 in K562-S cells, and no significant changes in the markers examined in K562-R cells.

Collectively, these findings define specific metabolic, redox, and autophagy-related features associated with the different imatinib sensitivity profiles of these K562-derived sublines. However, their functional contribution to resistance remains to be established. Further studies using additional CML models and functional approaches will be required to determine their mechanistic and therapeutic relevance.

## Figures and Tables

**Figure 1:**
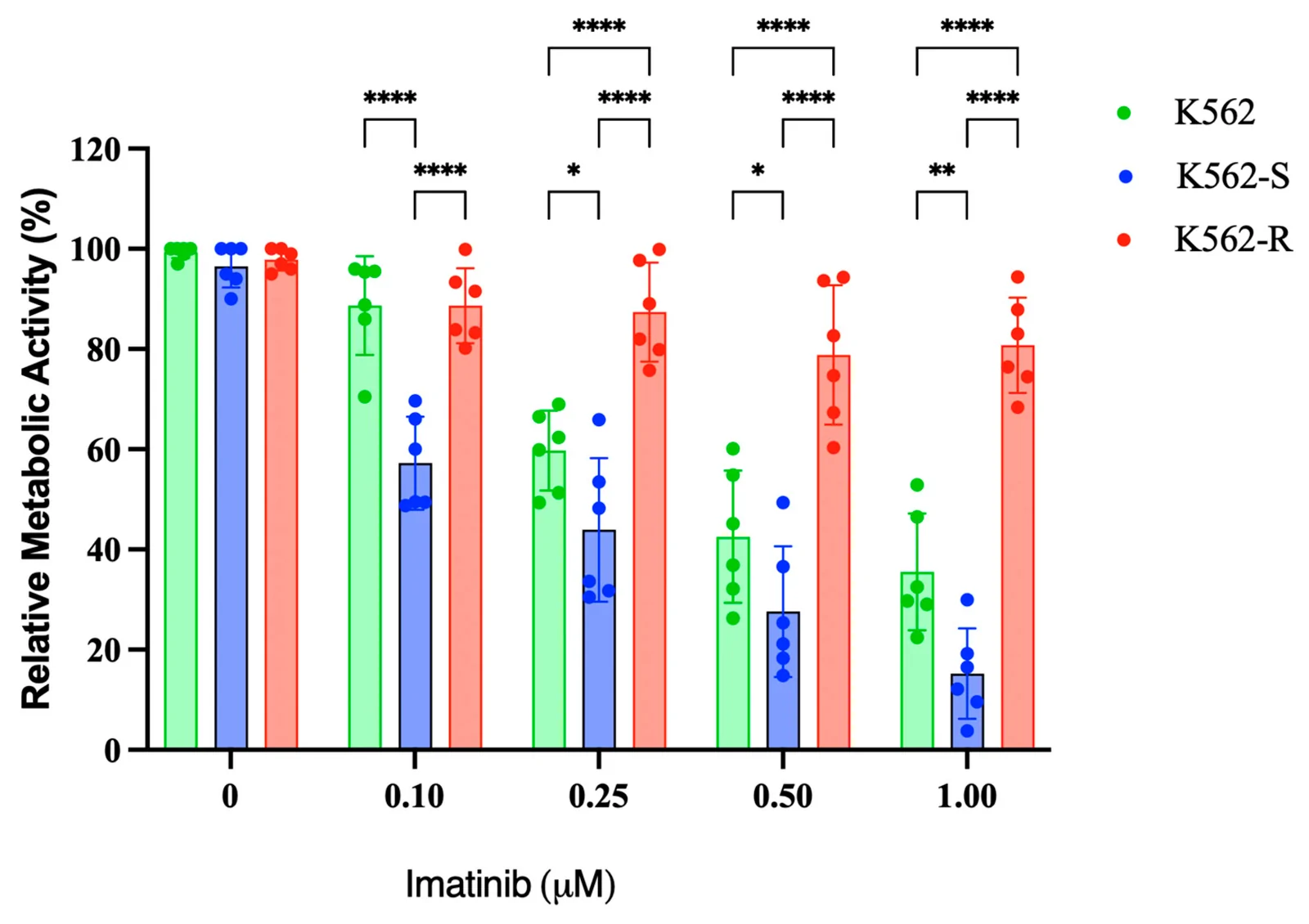
Imatinib differentially affects the metabolic activity of the K562, K562-S, and K562-R cell lines. K562, K562-S, and K562-R cells were treated with increasing concentrations of imatinib (0, 0.10, 0.25, 0.50, and 1.00 µM) for 72 h, and relative metabolic activity was assessed via the MTT assay. The values are expressed as percentages relative to the vehicle-treated control of each cell line (set at 100%) and are presented as the means ± SEMs (*n* = 6). Statistical significance was determined by two-way ANOVA followed by Bonferroni post hoc test for multiple comparisons between cell lines at each concentration. * *p* < 0.05, ** *p* < 0.01, **** *p* < 0.0001.

**Figure 2:**
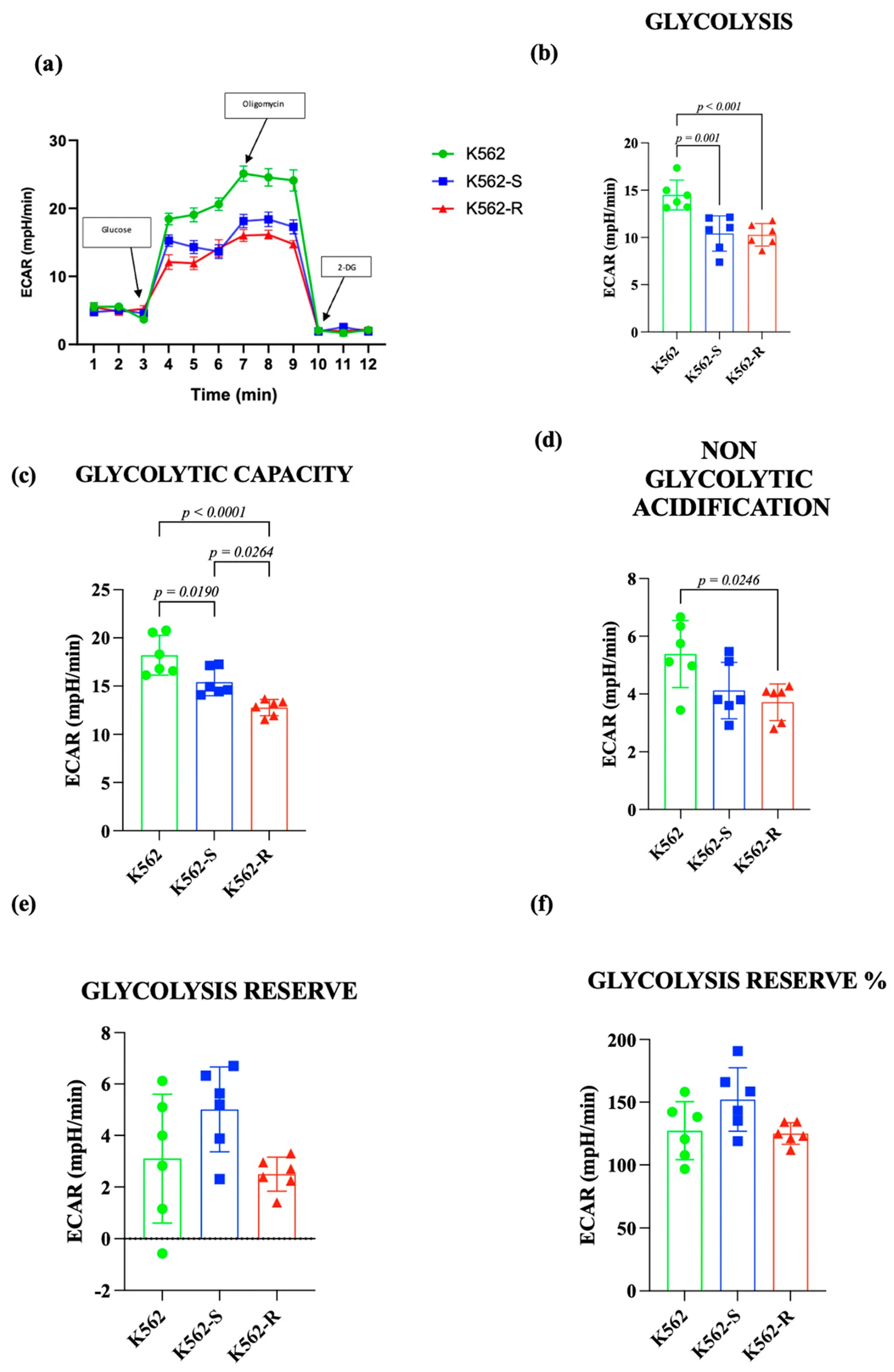
Reduced glycolytic activity in imatinib-sensitive and imatinib-resistant K562 cell sublines. (**a**) Real-time recording of the extracellular acidification rate (ECAR) across parental K562, imatinib-sensitive (K562-S), and imatinib-resistant (K562-R) cell lines. (**b**) Compared with parental K562 cells, both K562-S and K562-R cells exhibited significantly reduced glycolysis, with no significant difference between the two sublines. (**c**) Glycolytic capacity was significantly lower in both K562-S and K562-R cells than in K562 cells and was further reduced in K562-R cells compared with K562-S cells. (**d**) Non-glycolytic acidification was significantly lower in K562-R cells than in K562 cells. (**e**,**f**) No significant differences were observed in the glycolytic reserve or the glycolytic reserve% across the three cell lines. The data are presented as the means ± SEMs (*n* = 6 per group). Significance was assessed by one-way ANOVA followed by the Bonferroni post hoc correction.

**Figure 3:**
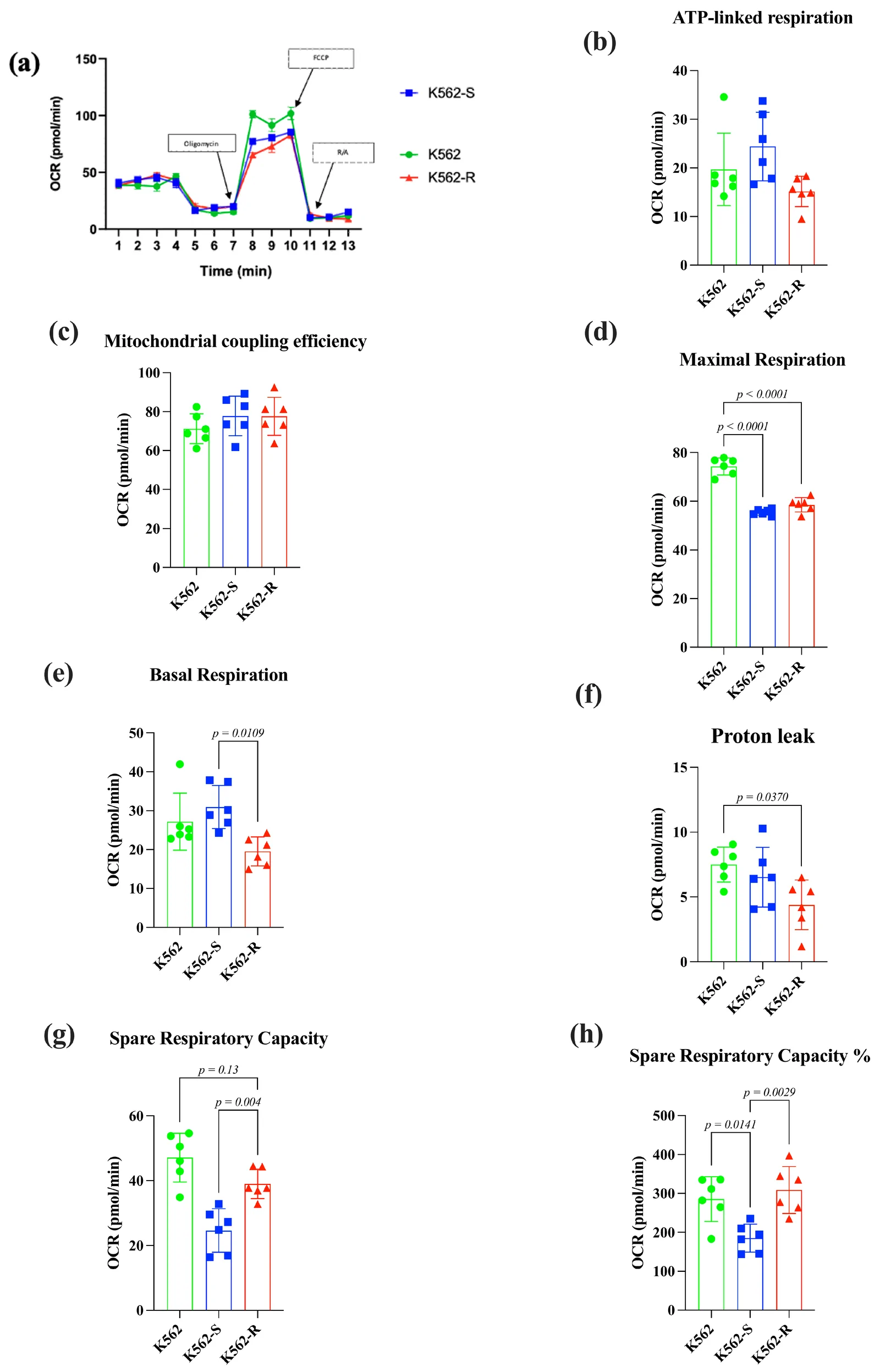
Differential mitochondrial respiratory profiles in imatinib-sensitive and imatinib-resistant K562 cells. (**a**) Real-time recording of the oxygen consumption rate (OCR) across parental K562, imatinib-sensitive (K562-S), and imatinib-resistant (K562-R) cell lines. (**b**,**c**) No significant differences in ATP-linked respiration or mitochondrial coupling efficiency were detected. (**d**) Maximal respiration was significantly lower in both K562-S and K562-R cells than in parental K562 cells. (**e**) Basal respiration was significantly lower in K562-R cells than in K562-S cells. (**f**) Proton leakage was significantly lower in K562-R cells than in K562 cells. (**g**,**h**) Spare respiratory capacity and spare respiratory capacity% were significantly lower in K562-S cells than in both K562 and K562-R cells, with K562-R cells showing greater spare capacity than K562-S cells. Data are presented as the means ± SEMs (*n* = 6 per group). Significance was assessed by one-way ANOVA followed by the Bonferroni post hoc correction.

**Figure 4:**
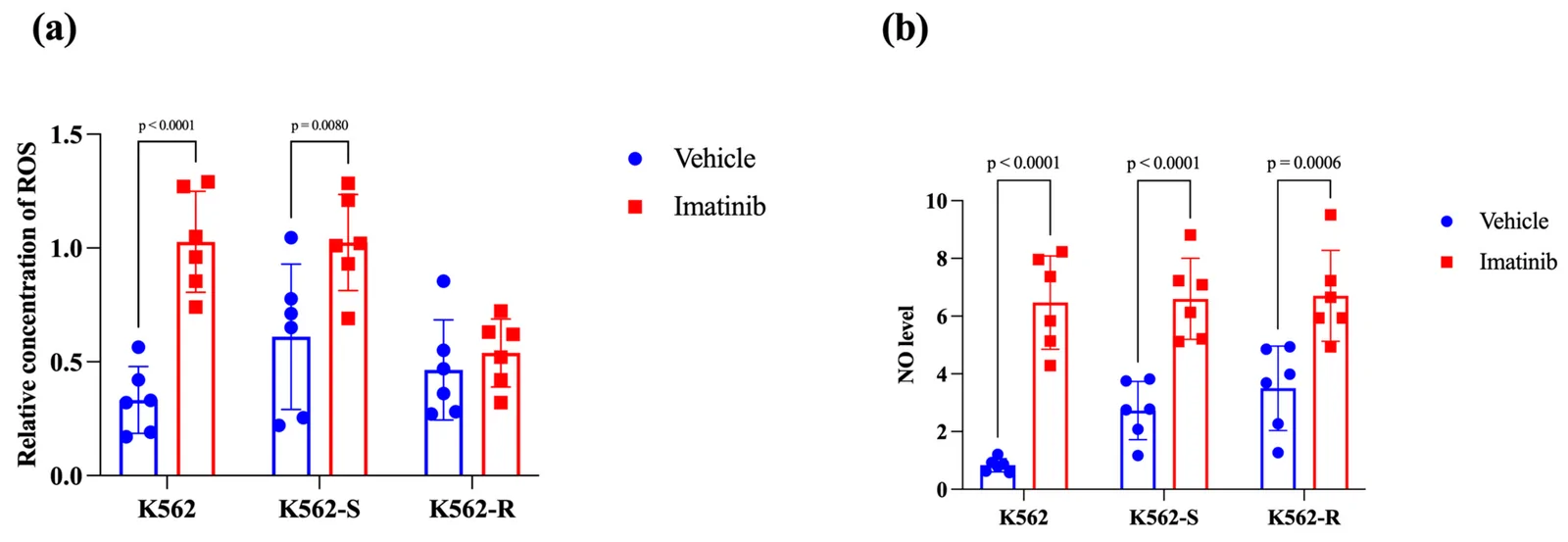
Imatinib differentially affects oxidative stress markers across CML cell sublines. K562, K562-S, and K562-R cells were treated for 72 h with imatinib (0.445 µM for K562, 0.137 µM for K562-S, and 1 µM for K562-R) or with vehicle. (**a**) Intracellular ROS levels were assessed via a redox-sensitive fluorescent probe. Compared with vehicle, imatinib significantly increased ROS levels in K562 and K562-S cells, whereas no significant change was observed in K562-R cells. (**b**) NO production was assessed indirectly via nitrite accumulation in the culture medium. Compared with vehicle, imatinib significantly increased NO levels in all three cell lines. The values are presented as the means ± SEMs (*n* = 6). Statistical significance was determined by two-way ANOVA followed by Bonferroni post hoc correction for multiple comparisons between vehicle- and imatinib-treated cells within each cell line.

**Figure 5:**
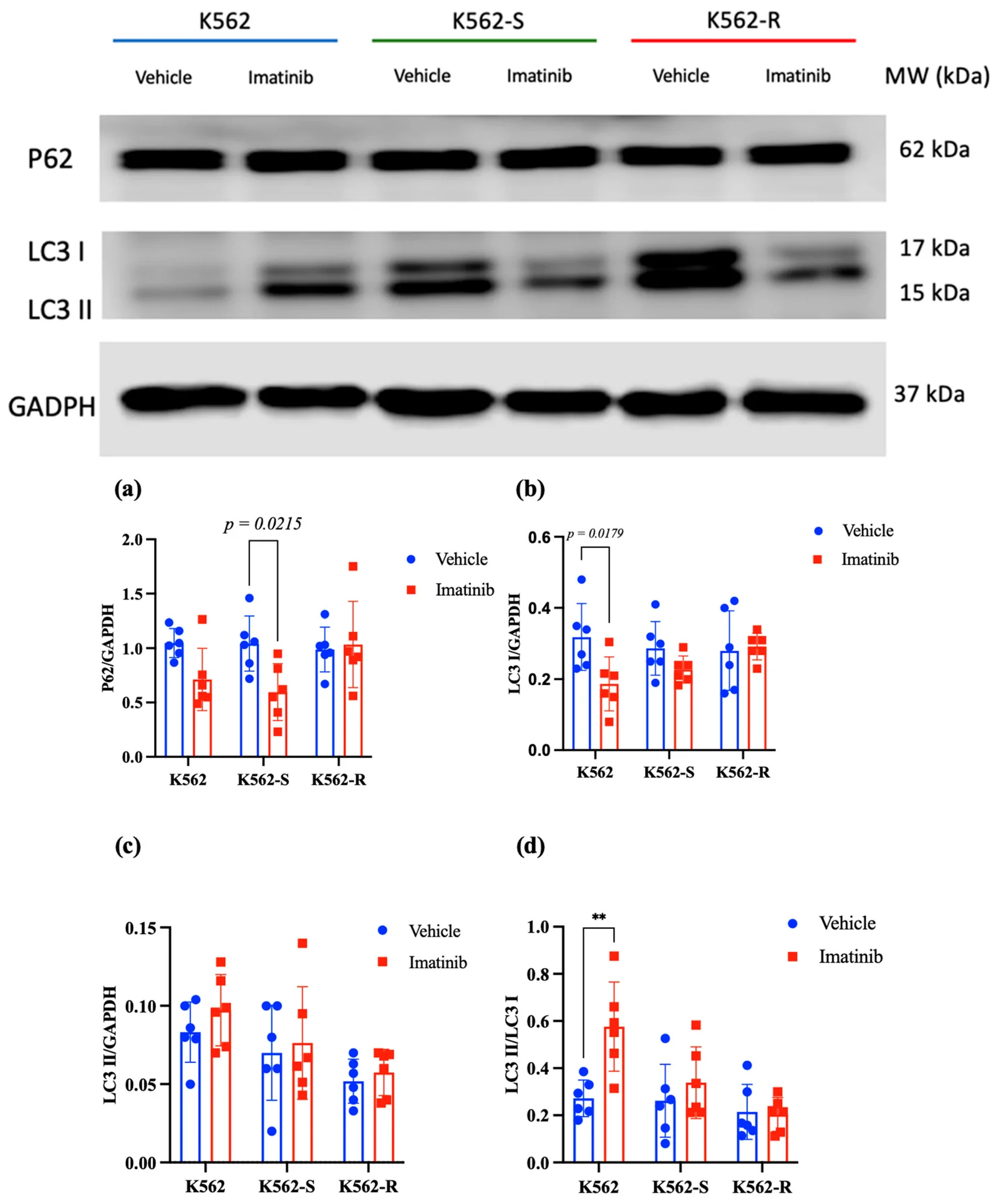
Imatinib differentially modulates autophagy markers in K562, K562-S, and K562-R cells. K562, K562-S, and K562-R cells were treated for 72 h with vehicle or imatinib (0.445 µM for K562, 0.137 µM for K562-S, and 1 µM for K562-R). Representative immunoblots of p62 (62 kDa), LC3-I (17 kDa), LC3-II (15 kDa), and GAPDH (37 kDa) are shown. Densitometric quantification of (**a**) p62/GAPDH, (**b**) LC3-I/GAPDH, (**c**) LC3-II/GAPDH, and (**d**) the LC3-II/LC3-I ratio is presented as the mean ± SEM (*n* = 6). Statistical significance was determined by two-way ANOVA followed by Bonferroni post hoc test for multiple comparisons between vehicle- and imatinib-treated cells within each cell line. **: *p* < 0.01.

## Data Availability

The raw data supporting the conclusions of this article will be made available by the authors on request.
